# Temporal rank of clinical characteristics and prognosis of anti‐*N*‐methyl‐d‐aspartate receptor encephalitis

**DOI:** 10.1002/brb3.2277

**Published:** 2021-07-07

**Authors:** Runnan Yang, Fenfen Ge, Jingwen Jiang, Yue Wang, Mengtong Wan, Wei Zhang

**Affiliations:** ^1^ Mental Health Center West China Hospital Sichuan University Chengdu Sichuan China; ^2^ West China Biomedical Big Data Center West China Hospital Sichuan University Chengdu Sichuan China; ^3^ Wuyuzhuang Honors College Sichuan University Chengdu Sichuan China

**Keywords:** anti‐*N*‐methyl‐d‐aspartate receptor encephalitis, diagnosis, electronic medical records, prognosis

## Abstract

**Objectives:**

Early recognition and intervention of patients with the anti‐*N*‐methyl‐d‐aspartate receptor (NMDAR) encephalitis are important to achieve a better prognosis. The study aims to summarize the real‐world perspectives of anti‐NMDAR encephalitis patients in China via electronic medical records (EMRs).

**Methods:**

Using EMRs of patients from 2013 to 2019 from West China Hospital in China, a retrospective research was conducted to demonstrate the temporary rank of clinical characteristics and disease prognosis of anti‐NMDAR encephalitis. The modified Rankin Scale (mRS) scores were used to divide the anti‐NMDAR‐encephalitis into two groups (poor prognosis vs. good prognosis). Chi‐square test and logistic regression were used to analyze factors associated with prognosis.

**Results:**

Here, 78 patients were included. The most common clinical characteristics are cognitive dysfunction (86.0%) and thought disorder (86.0%). Cognitive dysfunction, thought disorder, and seizures tended to appear soon after prodrome symptoms. Logistics analysis results showed that cognitive dysfunction (OR = 4.48, 95% CI = 1.09–18.47), the score of (GCS ≤ 8) (OR = 4.52, 95% CI = 1.18–17.32), positive antibodies in serum (OR = 4.89, 95% CI = 1.19–20.13) and delay immunotherapy (OR = 4.76, 95% CI = 1.79–12.60) were risk factors of poor clinical outcomes.

**Conclusions:**

There are two peaks in the development of autoimmune encephalitis (AE). The first peak is cognitive dysfunction, and the second peak is autonomic dysfunction. Cognitive dysfunction and GCS score ≤8 at admission, antibodies positive in serum, and delay immunotherapy were risk factors for a poor prognosis at discharge.

## Graphical abstract

Using electronic medical records (EMRs) of patients between 2013 to 2019 from West China Hospital in China, a retrospective research was conducted to demonstrate the temporary rank of clinical characteristics and disease prognosis of anti‐N‐methyl‐D‐aspartate receptor (NMDAR) encephalitis. We found that the most common clinical characteristics are cognitive dysfunction (86.0%) and thought disorder (86.0%). Logistics analysis results showed that cognitive dysfunction (OR=4.48, 95%, CI=1.09‐18.47), the score of (GCS≤8)(OR=4.52, 95%, CI=1.18‐17.32), positive antibodies in serum(OR=4.89, 95%CI=1.19‐20.13) and delay immunotherapy (OR=4.76, 95%, CI=1.79‐12.60) were risk factors of poor clinical outcomes.

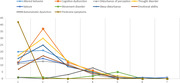

## 1 INTRODUCTION

Encephalitis is an inflammatory disease of the brain caused by an infectious pathogen or by autoimmune processes. Autoimmune encephalitis (AE) can be associated with specific autoantibodies, such as classical onconeuronal antibodies (e.g., anti‐Hu,Yo,Ri,Ma2,CV2),which targets intracellular antigens and are often related to underlying cancer. They can be associated with T‐cell‐mediated cytotoxicity (Bien et al., 2012). Generally speaking, onconeuronal antibodies were considered to be related with classical limbic encephalitis (LE). However, the antibodies against neuronal cell surface antigens were discovered in the studies of limbic encephalitis, referred to neuronal surface antibody syndromes (NSAS; Zuliani et al., 2019). In 2000, Bien et al. reported four patients with LE without tumor. In 2001, Buckley et al. found two patients with LE had voltage‐gated potassium channel (VGKC) antibody while their onconeuronal antibody was negative. Subsequent works identified that VGKC‐antibody‐associated encephalopathy is a common form of autoimmune, non‐paraneoplastic (Vincent et al., 2004) and reversible disease (Thieben et al., 2004). AE had gradually entered the public eye since the first case of anti‐*N*‐methyl‐d‐aspartate receptor (NMDAR) encephalitis was reported in 2007 (Dalmau et al., 2007). AE accounts for at least 20% of encephalitis (Granerod et al., 2013). Although the AE is rare, with an estimated incidence of 0.8/100,000 per year in the western population (Dubey et al., 2018), the influence of this disease in neurology and psychiatry is considered remarkable (Dalmau & Graus, 2018). Moreover, anti‐NMDAR encephalitis is the most common form of AE (Dubey et al., 2018). Given that patients with anti‐NMDAR encephalitis present a constellation of symptoms that are usually atypical and varied (Dalmau et al., 2008), this disease is difficult to be diagnosed at an early stage. Therefore, providing timely diagnosis and identified risk factors is very important (Vollmer & Mccarthy, 2016).

The anti‐NMDAR encephalitis usually progresses rapidly over days or weeks, usually starting with atypical psychiatric symptoms (e.g., alter mood, memory deficit or sleep disturbance) or prodrome symptoms (e.g., fever or headache). Dalmau's study found that only 23% of patients with anti‐NMDAR encephalitis were initially inspected by a neurologist, while 77% were first seen by a psychiatrist (Dalmau et al., 2008). Not handling anti‐NMDAR encephalitis timely can worsen psychiatric symptoms. In turn, it can lead to delay in correct diagnosis, which affects the identification by psychiatrists. Although previous researches have demonstrated that 81% of patients with anti‐NMDAR encephalitis have a good prognosis (Titulaer et al., 2013), 86% of patients will have long‐term neurological deficits (e.g., fatigue and emotional lability; Yeshokumar et al., 2017) and 5−11% of the anti‐NMDAR encephalitis will die (Chi et al., 2017). Thus, a comprehensive understanding of what factors may affect the prognosis of anti‐NMDAR encephalitis can potentially influence treatment regimens and is essential in offering a beneficial perspective to clinicians, patients, and family members.

Capturing and using clinical information to ensure a safe, high quality, and sustainable healthcare service is vital. Information from electronic medical records (EMRs) has been important to decision‐making on the disease (Fennelly et al., 2020). EMRs can provide real‐world clinical information on disease development, progression, and intervention strategies. Effectively using the EMRs contributes to reduce repetition of tests and work and promotes the safety and quality of healthcare provided (Castillo et al., 2010; O'Donnell et al., 2018).

Thus, this survey aimed to answer the following questions based on EMRs. What are the frequency and temporal rank of anti‐NMDAR encephalitis? What factors can be used to predict the prognosis? Answers from the above questions can be used as real‐world evidence to timely identify and effectively manage anti‐NMDAR encephalitis.

## 2 METHODS

### 2.1 Data source

This research has attained approval from the Ethics Committee of the West China Hospital, Sichuan University. We analyzed the data from the EMRs of West China Hospital. The hospital has a large volume of patient‐level data, which provides a platform for accomplishing a retrospective database research among patients with anti‐NMDAR encephalitis. During the research, patients’ personal information was kept confidential.

### 2.2 Procedure

**FIGURE 1:**
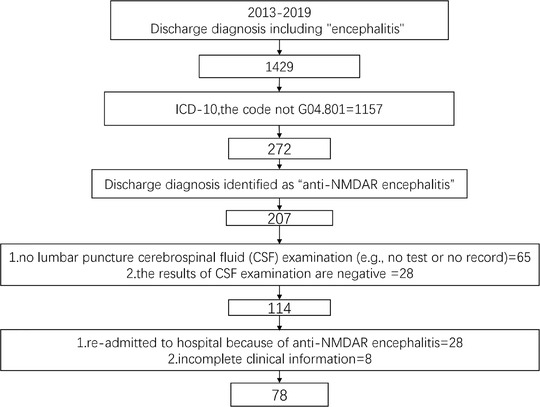
The process of data extraction

Data between January 1, 2013, and December 30, 2019, were extracted, mainly including information on patient clinical features, diagnosis, and detailed treatment‐related strategies in the period of hospitalization. Inclusion criteria were: (1) primary discharge diagnosis as anti‐NMDAR encephalitis; (2) first‐episode drug‐naïve anti‐NMDAR encephalitis. Exclusion criteria included were: (1) no lumbar puncture cerebrospinal fluid (CSF) examination (e.g., no test or no record); (2) negative for NMDAR antibodies in CSF examination (Gresa‐Arribas et al., 2014); (3) re‐admitted to hospital because of anti‐NMDAR encephalitis; (4) incomplete clinical information. The process of data extraction is shown in Figure 1.

### 2.3 Study variables

The demographic (gender and age) is directly extracted from EMRs. Chart review was conducted to gain data regarding incidence trend and clinical characteristics at admission. The incidence trend was calculated by the annual number of diagnosed cases of anti‐NMDAR encephalitis and the time for primary diagnosis (no‐anti‐NMDAR encephalitis) to diagnosis correcting (anti‐NMDAR encephalitis). The clinical characteristics include the score of Glasgow coma scale (GCS), altered behavior, cognitive dysfunction, disturbance of perception, thought disorder, seizure, movement disorder, sleep disturbance, emotional lability, autonomic dysfunction, and viral prodrome. The GCS score was used to assess the level of consciousness of patients at admission. Autonomic dysfunction was detected by sustained atrial tachycardia or bradycardia, orthostatic hypotension (≥20 mmHg fall in systolic pressure or ≥10 mmHg falls in diastolic pressure within 3 min of standing), hyperhidrosis, persistently labile blood pressure, ventricular tachycardia, or cardiac asystole (Dubey et al., 2017). Prodromal symptoms were defined as patients presenting the following symptoms: rhinorrhea, sore throat, diarrhea, and fever (Dubey et al., 2017). The time of “interval to immunotherapy” is defined as the time from admission to start using immunotherapy.

Antibody tests for all patients were accomplished in the same laboratory of Peking Union Medical College Hospital, China. The titers of serum antibodies were considered as weakly positive (1:10), positive (1:32 to 1:100), and strongly positive (1:320), respectively. CSF antibody titers were defined as weakly positive (1:1), positive (1:3.2 to 1:10), and strongly positive (≥1:32) (Gu et al., 2019).

The results of other laboratory tests (electroencephalography [EEG], brain magnetic resonance imaging [MRI], chest and abdomen computerized tomography [CT]) were directly extracted from the EMRs. We judged whether the patients have tumors according to the results of the chest and abdomen CT.

The process of treatment (treatment patterns of first‐line and second‐line, intensive care unit [ICU] admission, mechanical ventilation) was reviewed and documented by two authors. Treatment patterns can be divided as first‐line treatment (high‐dose steroids, plasma exchange [PE], and/or IV immunoglobulin [IVIG]) and second‐line treatment (rituximab [RTX] and/or cyclophosphamide [CP]; Titulaer et al., 2013; Yeshokumar et al., 2017).

We used the modified Rankin Scale (mRS) to divide the anti‐NMDAR encephalitis into good prognosis (mRS scores of 0–2) and poor prognosis (mRS scores of 3–6) at the time of discharge from hospital (Gresa‐Arribas et al., 2014).

### 2.4 Statistics

This research was primarily descriptive, and as such, data were summarized and presented as percentages or medians with interquartile ranges as appropriate for the data types (continuous variables and categorical variables). Difference between groups (good prognosis vs. poor prognosis) is identified by the Chi‐square test for categorical variables. Estimated coefficients (βs) and odds ratios (ORs) were calculated via logistic regression. *p *< 0.05 was considered as statistical significance. All statistical analyses were performed using R‐studio 3.6.

## 3 RESULT

### 3.1 Demographic characteristics and incidence trends

A search of EMRs yielded 1429 patients with a primary discharge diagnosis of encephalitis, of which 78 was considered eligible anti‐NMDAR encephalitis cases. Of the 78 patients, females (42, 53.85%) outnumbered males (36, 46.15%). With a median age of 29 at admission, the youngest patient was 14 years old and the oldest was 65 years old.

**FIGURE 2:**
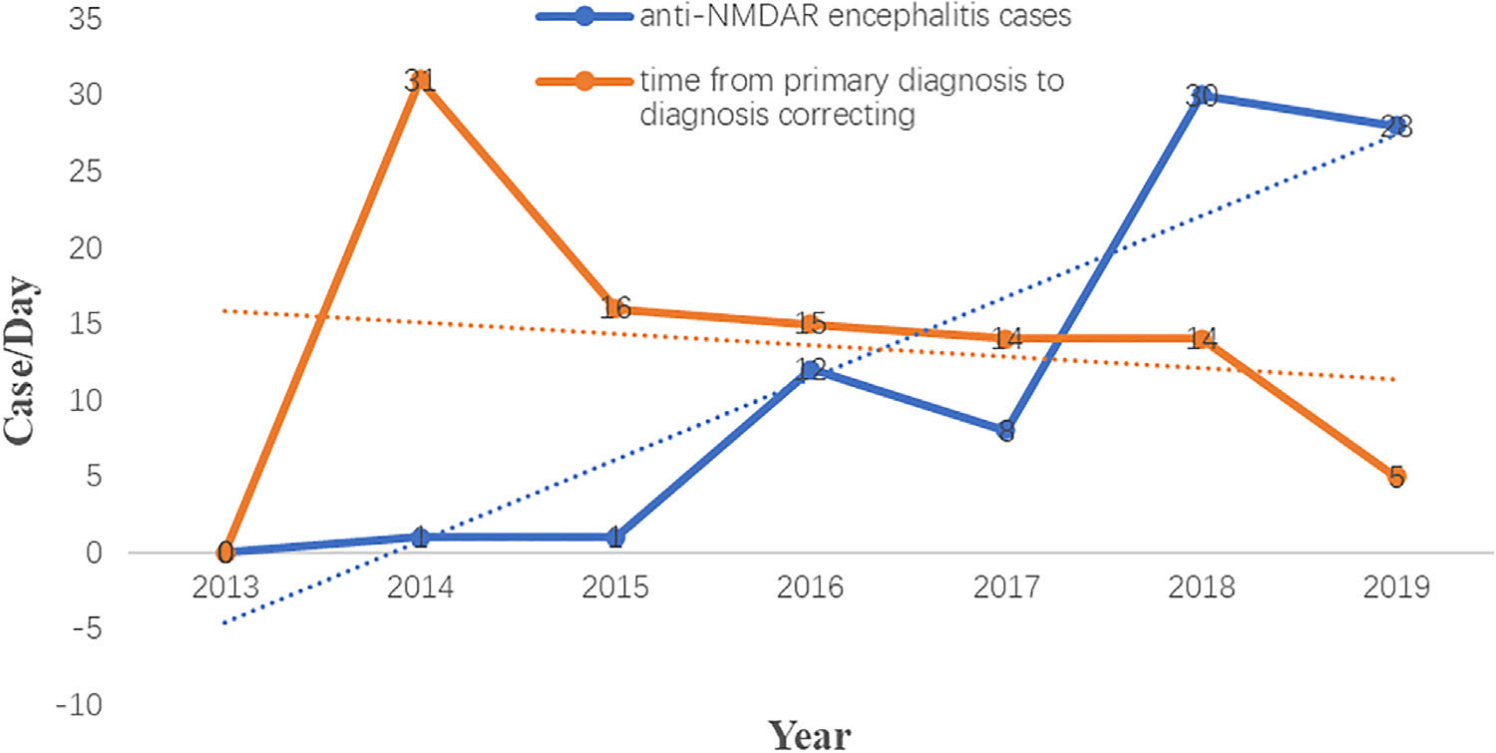
The annual number of diagnosed cases and corrected diagnosis of anti‐NMDAR encephalitis. The blue line illustrates change in the number of anti‐NMDAR encephalitis cases diagnosed per year. The red line illustrates the changes in time from primary diagnosis to diagnosis correcting

Of the 78 patients, 0 was diagnosed in 2013, accounting for 0% of all patients; 2 in 2014, accounting for 1.7%; 2 in 2015, accounting for 1.7% and 9 in 2016, accounting for 7.4%. There were 16 cases in 2017 and 36 cases in 2018. In 2019, there were 56 cases, accounting for 46.3%. As shown in Figure 2, the annual number of diagnosed cases of anti‐NMDAR encephalitis trended upward. Of the 78 patients, 14 were misdiagnosed as no‐AE (e.g., acute and transient psychotic disorder, schizophrenia, manic episode, and major depressive disorder) at admission, accounting for 17.95%.

### 3.2 Clinical characteristics

**FIGURE 3:**
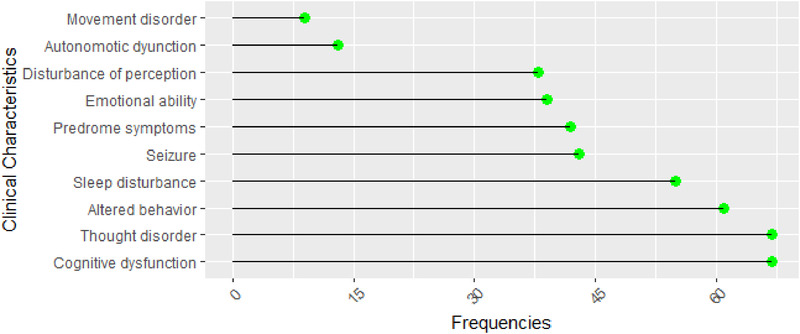
Clinical characteristics of anti‐NMDAR encephalitis patients at admission. Data extracted from the EMRs of West China Hospital during January 1, 2013 and December 30, 2019

The temporal sequence where each clinical characteristic was first reported in EMRs was coded numerically as ranks 1, 2, 3, 4, 5, etc. (1 = first, 2 = second, 3 = third, 4 = fourth, 5 = fifth, etc.). Clinical characteristics were recorded in EMRs. If the clinical features were not reported in EMRs, it was scored as “absent.” In turn, it was scored as “present.” If two clinical features occur at the same time, they were coded in the same temporal sequence. If the clinical features were not reported in EMRs, it was scored as “absent.” The frequency of clinical features was presented in Figure 3. The most commonly observed clinical characteristics were cognitive dysfunction (67, 85.90%), thought disorder (67, 85.90%), altered behavior (61, 78.21%), sleep disturbance (55, 70.51%), seizure (43, 55.13%), prodrome symptoms (42, 53.85%), emotional ability (39, 50.00%), disturbance of perception (38, 48.72%), autonomic dysfunction (13, 16.67%), and movement disorder (9, 11.54%).

**FIGURE 4:**
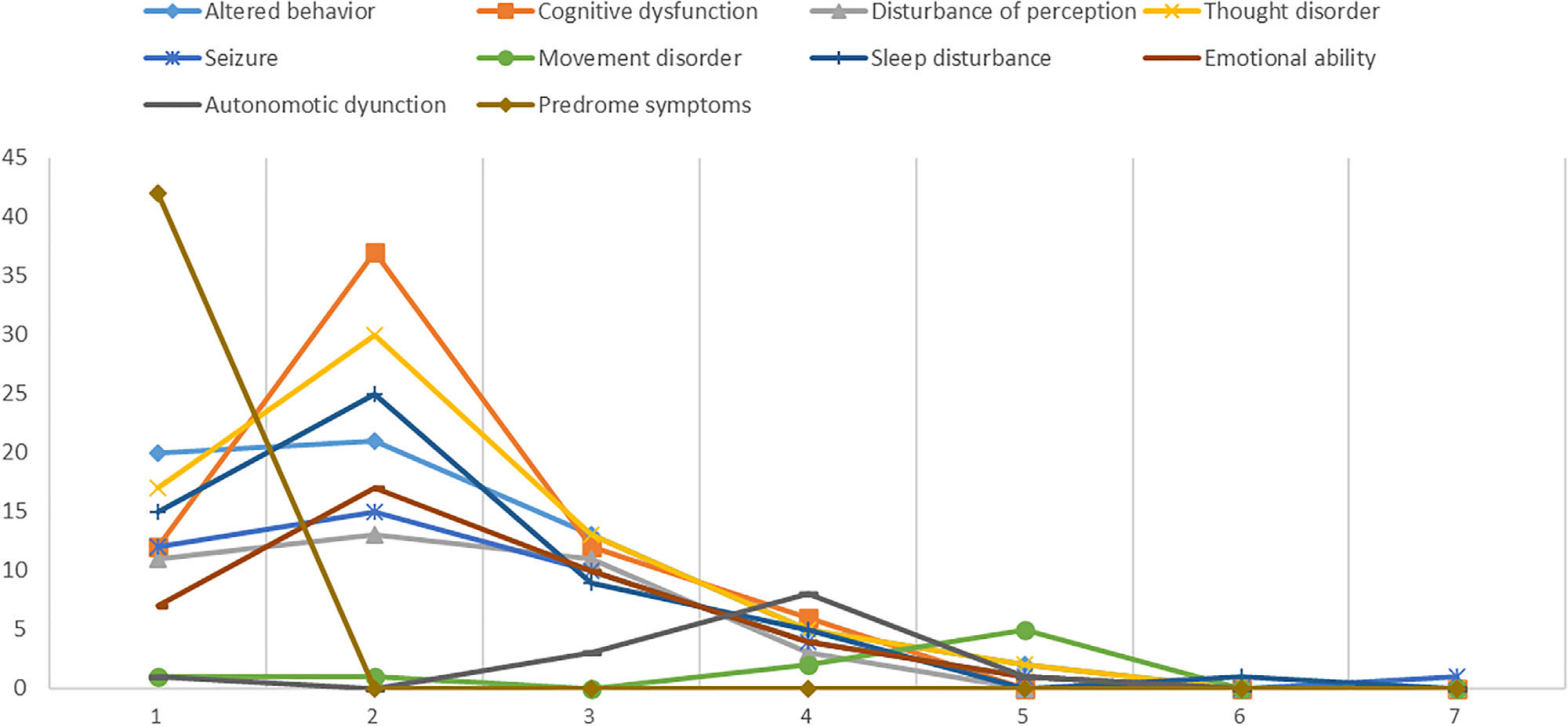
Temporal sequence of the first appearance. The *x*‐axis represents the temporal rank of each clinical characteristic. The *y*‐axis represents the frequency that each clinical characteristic was sorted into different ranks

Figure 4 illustrates that patients whose disease process was related to clinical characteristics shows prodrome symptoms that dominate the earliest phase of the anti‐NMDAR encephalitis phase. Cognitive dysfunction, thought disorder, and seizures tended to appear soon after the prodrome symptoms. Autonomic dysfunction and movement disorder suggested a secondary and a third peak, respectively.

### 3.3 Univariate analysis

Among 78 anti‐NMDAR encephalitis patients, 33 (42.30%) had a favorable prognosis and 45 (57.70%) patients had a poor prognosis at discharge. There were significant variables including cognitive dysfunction (*p *= 0.03), seizure (*p *= 0.05) and movement disorder (*p *= 0.04), antibody‐positive in serum (*p *= 0.02), utilization of mechanical ventilation (*p *= 0.04), GCS score ≤8 (*p *= 0.02) and interval to immunotherapy (*p *= 0.00) between groups. The details are shown in Table 1.

**TABLE 1:** Results from the univariate analysis of the two groups

	Prognosis			
Variable	Good^a^ (*n* = 33)	Poor^b^ (*n* = 45)	*X* ^2^	*p*
Demographic				
Age			0.00	0.97
<29	16 (48.48%)	22 (48.89%)		
≥29	17 (51.52%)	23 (51.11%)		
Sex			2.21	0.17
Male	12 (36.36%)	24 (53.33%)		
Female	21 (63.64%)	21 (46.67%)		
Clinical characteristics				
Altered behavior			2.43	0.12
Yes	23 (69.70%)	38 (84.44%)		
No	10 (30.30%)	7 (15.56%)		
Cognitive dysfunction			4.86	0.03
Yes	25 (75.76%)	42 (93.33%)		
No	8 (24.24%)	3 (6.67%)		
Disturbance of perception			0.24	0.62
Yes	15 (45.45%)	23 (51.11%)		
No	18 (54.55%)	22 (48.89%)		
Thought disorder			0.79	0.38
Yes	27 (81.82%)	40 (88.89%)		
No	6 (18.18%)	5 (11.11%)		
Seizure			3.73	0.05
Yes	14 (42.42%)	29 (64.44%)		
No	19 (57.58%)	16 (35.56%)		
Movement disorder			4.06	0.04
Yes	1 (3.03%)	8 (17.78%)		
No	32 (96.97%)	37 (82.22%)		
Sleep disturbance			1.88	0.17
Yes	26 (78.79%)	29 (64.44%)		
No	7 (21.21%)	16 (35.56%)		
Emotional ability			0.47	0.49
Yes	15 (45.45%)	24 (53.33%)		
No	18 (54.55%)	21 (46.67%)		
Autonomic dysfunction			0.1	0.76
Yes	6 (18.18%)	7 (15.56%)		
No	27 (81.82%)	38 (84.44%)		
Prodrome symptoms			0.13	0.72
Yes	17 (51.52%)	25 (55.56%)		
No	16 (48.48%)	20 (44.44%)		
Antibodies titers				
CSF			2.87	0.24
Weakly positive	3 (9.09%)	2 (4.44%)		
Positive	11 (33.33%)	9 (20.00%)		
Strongly positive	19 (57.58%)	34 (75.56%)		
Serum			9.45	0.02
Negative	24 (72.73%)	18 (40.00%)		
Weakly positive	5 (15.15%)	9 (20.00%)		
Positive	3 (9.09%)	11 (24.44%)		
Strongly positive	1 (3.03%)	7 (15.56%)		
Auxiliary examination				
GCS			5.42	0.02
≤8	3 (9.09%)	14 (31.11%)		
>8	30 (90.91%)	31 (68.89%)		
MRI			1.09	0.58
Normal	11 (33.33%)	20 (44.44%)		
Abnormal	20 (60.61%)	22 (48.89%)		
Not report	2 (6.06%)	3 (6.67%)		
EEG			0.31	0.86
Normal	5 (15.15%)	5 (11.11%)		
Abnormal	19 (57.58%)	28 (62.22%)		
Not report	9 (27.27%)	12 (26.67%)		
Tumor			3.25	0.07
Yes	1 (3.03%)	7 (15.56%)		
No	32 (96.97%)	38 (84.44%)		
Therapy strategies				
Mechanical ventilation			4.06	0.04
Yes	1 (3.03%)	8 (17.78%)		
No	32 (96.97%)	37 (82.22%)		
Transfer to ICU			3.09	0.08
Yes	0 (0.00%)	4 (8.89%)		
No	33 (100.0%)	41 (91.11%)		
Interval to immunotherapy^c^			10.4	0
<5 days	20 (60.61%)	11 (24.44%)		
≥5 days	13 (39.39%)	34 (75.56%)		

^a^ Modified Rankin Scale (mRS) scores of 0–2.

^b^ Modified Rankin Scale (mRS) scores of 3–6.

^c^ The time from admission to start of immunotherapy (median, 5 days).

Abbreviations: CSF = cerebrospinal fluid; EEG = electroencephalography; GCS = Glasgow Coma Scale; ICU = intensive care unit; MRI = magnetic resonance imaging.

### 3.4 Logistics analysis of a poor prognosis

The logistics analysis results demonstrated that cognitive dysfunction (OR = 4.48, 95%, CI = 1.09–18.47), positive serum antibody (OR = 4.89, 95% CI = 1.19–20.13), strongly positive serum antibody (OR = 9.33, 95% CI = 1.05–82.78), score of GCS ≤8 (OR = 4.52, 95% CI = 1.18–17.32) and delay immunotherapy (OR = 4.76, 95% CI = 1.79–12.60) were risk factors of poor prognosis. There is no significant association in other factors, such as movement disorder and utilization of mechanical ventilation, and a poor prognosis. The details are shown in Table 2.

**TABLE 2:** Logistics analysis of risk factors associated with poor clinical outcomes

Variable	Case (good^a^/total)	OR, 95% CI
Demographic		
Age		
<29	16 (48.48%)	Ref.
≥29	17 (51.52%)	0.98 (0.40–2.42)
Sex		
Male	12 (36.36%)	Ref.
Female	21 (63.64%)	0.50 (0.20–1.25)
Clinical characteristics		
Altered behavior		
Yes	23 (69.70%)	Ref.
No	10 (30.30%)	2.36 (0.79–7.06)
Cognitive dysfunction		
Yes	25 (75.76%)	Ref.
No	8 (24.24%)	4.48 (1.09–18.47)
Disturbance of perception		
Yes	15 (45.45%)	Ref.
No	18 (54.55%)	1.26 (0.51–3.09)
Thought disorder		
Yes	27 (81.82%)	Ref.
No	6 (18.18%)	1.78 (0.49–6.42)
Seizure		
Yes	14 (42.42%)	Ref.
No	19 (57.58%)	2.46 (0.98–6.18)
Movement disorder		
Yes	1 (3.03%)	Ref.
No	32 (96.97%)	6.92 (0.82–58.34)
Sleep disturbance		
Yes	26 (78.79%)	Ref.
No	7 (21.21%)	0.49 (0.17–1.37)
Emotional ability		
Yes	15 (45.45%)	Ref.
No	18 (54.55%)	1.37 (0.56–3.38)
Autonomic dysfunction		
Yes	6 (18.18%)	Ref.
No	27 (81.82%)	0.83 (0.25–2.74)
Prodrome symptoms		
Yes	17 (51.52%)	Ref.
No	16 (48.48%)	1.18 (0.48–2.90)
Antibodies titers		
CSF		
Weakly positive	3 (9.09%)	Ref.
Positive	11 (33.33%)	1.23 (0.17–9.02)
Strongly positive	19 (57.58%)	2.68 (0.41–17.51)
Serum		
Negative	24 (72.73%)	Ref.
Weakly positive	5 (15.15%)	2.40 (0.69–8.40)
Positive	3 (9.09%)	4.89 (1.19–20.13)
Strongly positive	1 (3.03%)	9.33 (1.05–82.78)
Auxiliary examination		
GCS		
≤8	3 (9.09%)	Ref.
>8	30 (90.91%)	4.52 (1.18–17.32)
MRI		
Normal	11 (33.33%)	Ref.
Abnormal	20 (60.61%)	0.61 (0.23–1.57)
Not report	2 (6.06%)	0.83 (0.12–5.71)
EEG		
Normal	5 (15.15%)	Ref.
Abnormal	19 (57.58%)	1.47 (0.38–5.80)
Not report	9 (27.27%)	1.33 (0.29–6.04)
Tumor		
Yes	1 (3.03%)	Ref.
No	32 (96.97%)	5.90 (0.69–50.48)
Therapy strategies		
Mechanical ventilation		
Yes	1 (3.03%)	Ref.
No	32 (96.97%)	6.92 (0.82–58.34)
Transfer to ICU		
Yes	0 (0.00%)	Ref.
No	33 (100.00%)	1300260257 (0–)
Interval to immunotherapy^b^		
<5 days	20 (60.61%)	Ref.
≥5 days	13 (39.39%)	4.76 (1.79–12.60)

^a^ Modified Rankin Scale (mRS) scores of 0–2.

^b^ The time from admission to start of immunotherapy (median, 5 days).

Abbreviations: CSF = cerebrospinal fluid; EEG = electroencephalography; GCS = Glasgow Coma Scale; ICU = intensive care unit; MRI = magnetic resonance imaging.

## 4 DISCUSSION

### 4.1 Demographic and incidence trend of anti‐NMDAR encephalitis patients

Previous studies found that nearly 80% of AE are seen in women, of which half of the cases occurred in women above the age of 18 (Dalmau et al., 2019). However, in the current research, the sex ratio for anti‐NMDAR encephalitis was nearly balanced. Previous researchers have found that the median age of anti‐NMDAR encephalitis patients was between 21 and 28 (Chi et al., 2017; Titulaer et al., 2013), while in this research the median age is 29. This may have contributed to our data bias. We collected data only from West China Hospital, and most children and adolescents may choose to go to a specialist hospital (e.g., West China Women's and Children's Hospital). From 2013 to 2019, the number of diagnosed anti‐NMDAR encephalitis cases has increased annually, which may be associated with the antibodies' detection methods. The time for primary diagnosis (no‐anti‐NMDAR encephalitis) to diagnosis correcting (anti‐NMDAR encephalitis) shows a downward trend, which may be associated with increased awareness of anti‐NMDAR encephalitis among psychiatrists and neurologists.

### 4.2 Frequency and temporal rank of anti‐NMDAR encephalitis patients

In 70% of patients, anti‐NMDAR encephalitis starts with prodromal symptoms (Dalmau et al., 2011; Luca et al., 2011).We found that 54% of patients have represented prodromal symptoms that appeared earlier relative to other symptoms. The prodromal infection may be regarded as the antigenic trigger for the inflation of anti‐NMDAR‐specific lymphocytes by molecular mimicry (Peery et al., 2012). The temporal rank of clinical characteristics demonstrated in this research is consistent with Irani's research (Irani et al., 2010), which showed that psychiatric and cognitive disorders appeared at an early stage. While in Gurrera's study (Gurrera, 2019), the present sample size collected from EMRs is much more extensive. Sleep disturbance may play the role as a bridge symptom in the progression of the disease. After the sleep disturbance, the second peak (autonomic dysfunction) and the third peak (movement disorder) appeared. Also, Blattner's study found that untreated sleep complaints may have adversely influenced the automatic and cognitive functions (Blattner & Day, 2020).

At present, there is scarce number of studies systematically assessing sleep disturbance in people with autoimmune encephalitis and most of them are based on case reports not focused on sleep (Muñoz‐Lopetegi et al., 2020). However, sleep disturbance is an essential part of anti‐NMDAR encephalitis and has relevant clinical implications. It is usually severe and persists beyond the acute phase of the disease, affecting the process of recovery and quality of the patients’ life (Ariño et al., 2020). Therefore, an adequate management for sleep disturbance may help in both diagnosis and overall recovery.

### 4.3 Factors for the prognosis of anti‐NMDAR encephalitis patients

In this research, we reported 33 patients who represent a good prognosis and evaluated the risk factors of poor prognosis. The good outcomes of anti‐NMDAR encephalitis in this study was 42.30%, which was much higher than 41% in the United States (Singh et al., 2015) and 38.3% in China (Mo et al., 2020). The variability may be explained by the differential medical model in different regions. Results showed that patients with cognitive dysfunction (95% CI = 1.09–18.47), seizure (95% CI = 0.98–6.18), movement disorder (95% CI = 0.82–58.34), and GCS ≤ 8 (95% CI = 1.18–17.32) were risk factors for poor clinical outcomes, while seizure and movement disorder have not achieved significant differences in logistics analysis. It is cautious that the CI is relatively wider in this study. There may still be enough precision to make decisions about factors of prognosis, as the width of the CI for an individual study depends to a large extent on the sample size (Julian Higgins, 2020) and our study have a smaller one. Some studies have found that the scores of GCS ≤8 can be regarded as a risk factor for a poor short‐term (at discharge) and or long‐term (3 months and 6 months after the discharge) clinical outcome (Chi et al., 2017; Mo et al., 2020). The most common reasons for ICU admission were seizure and altered mental state (Hacohen et al., 2013). Moreover, patients who were transferred to ICU often had a poor clinical outcome despite using timely immunotherapy (Mittal et al., 2016). In this study, most patients represent seizure and movement disorders during disease progression instead of the initial stage. Thus, timely diagnosis and immunotherapy is a critical prognostic factor (De Montmollin et al., 2017; Titulaer et al., 2013). The starting of immunotherapy as soon as possible can hinder disease progression and decrease the transfer rate to the ICU and promote a good clinical outcome. An unexpected finding was that positive and strongly positive titers in serum correlate with worse clinical outcome instead of titers in CSF. Gresa‐Arribas's study found an association between high titer in CSF and poor outcome and the decrease of titers in CSF correlated with better clinical outcomes (Gresa‐Arribas et al., 2014). All patients with “definite” anti‐NMDAR encephalitis have antibodies in CSF, while 14−30% of patients’ antibodies in the serum are negative (Gresa‐Arribas et al., 2014; Leypoldt et al., 2015; Newman et al., 2016). Thus, the examination of titers in serum plays an essential role in the process of treatment.

### 4.4 Clinical implications

Anti‐NMDAR encephalitis increase the economic burden on both patients and society (Cohen et al., 2019), but the management of anti‐NMDAR encephalitis patients (e.g., delays in diagnosis and transportation to specialized centers) in China is still poor. Patients with “red flag symptoms” (e.g., prodromal symptoms, acute or subacute psychiatric symptoms, and seizure; Pollak et al., 2020) should be handled urgently and undergo antibodies examination. Furthermore, the CSF antibody examination should be considered in the initial diagnostic testing. Examination of only the serum is insufficient, although serum testing is more sensitive for several specific antibodies (Leypoldt et al., 2015) and titers in serum are related to a clinical outcome at discharge. The risk factors of poor clinical outcomes can help clinicians evaluate the possible prognosis at the early stage and provide early monitoring and supportive treatment strategies. Besides, clinicians can interpret the possible clinical outcomes for patients and patients’ family members to promote the therapeutic relationship.

### 4.5 Strengths and limitations

The current study has some potential strengths, which mainly includes: (1) the data is directly extract from the EMRs. Real‐world evidence has the potential to offer useful information about the use and prognosis of a given clinical treatment within a setting most relevant to routine clinical practice in China; (2) we only included initial anti‐NMDAR encephalitis patients so the effect of the medication on anti‐NMDAR encephalitis was ruled out. However, our study had some limitations, including (1) the sample size of the current study was insufficient. (2) Patients with anti‐NMDAR encephalitis coming from only one specific hospital might limit the generalizability of our findings; (3) we only included “definite” anti‐NMDAR encephalitis patients whose anti‐NMDAR was detected in the CSF in this study. Probable anti‐NMDAR encephalitis was not included. (4) We only evaluated the prognosis at discharge, and cannot rule out the patients who were relapsed or dead. There is a need for prospective and multicenter trails in the future. (5) In the retrospective study, for the clinical symptoms that if it did not appear in the EMR was scored as “absent” (Gurrera, 2019), ignoring the probably missing data.

## 5 CONCLUSION

In summary, we analyzed the temporary rank of clinical characteristics and risk factors for prognosis among anti‐NMDAR encephalitis. Although the spectrum of features at admission and course of anti‐NMDAR encephalitis is wide, new psychiatric symptoms and accompanied prodromal symptoms can be regarded as “red flag” for anti‐NMDAR encephalitis. Cognitive dysfunction, antibodies titers in serum, GCS scores (≤8), and interval to immunotherapy (≥5 days) were risk factors for a poor prognosis.

## AUTHOR CONTRIBUTIONS

Wei Zhang conceived the study, contributed to the planning, draft, revision of the manuscript. Fenfen Ge, Runnan Yang, and Yue Wang wrote the first draft of this paper. Fenfen Ge and Jingwen Jiang analyzed and interpreted the data. Fenfen Ge and Mengtong Wan revised the draft critically for important intellectual content. All authors contributed to the revision of the manuscript. All authors gave the final approval of the version to be published.

### PEER REVIEW

The peer review history for this article is available at https://publons.com/publon/10.1002/brb3.2277.

## Data Availability

The original contributions presented in the study can be acquired from corresponding author.
